# Association of recurrent venous thromboembolism and circulating microRNAs

**DOI:** 10.1186/s13148-019-0627-z

**Published:** 2019-02-13

**Authors:** Xiao Wang, Kristina Sundquist, Peter J. Svensson, Hamideh Rastkhani, Karolina Palmér, Ashfaque A. Memon, Jan Sundquist, Bengt Zöller

**Affiliations:** 1 Center for Primary Health Care Research, Wallenberglaboratoriet, Lund University/Region Skåne, Inga-Marie Nilssons gata 53, plan 6, Box 50332, 202 13 Malmö, Sweden; 2 Department of Coagulation Disorders, Lund University, Malmö, University Hospital, Malmö, Sweden; 3 Department of Family Medicine and Community Health, Department of Population Health Science and Policy Icahn School of Medicine at Mount Sinai, New York, USA; 4 Center for Community-based Healthcare Research and Education (CoHRE), Department of Functional Pathology, School of Medicine, Shimane University, Matsue, Japan

**Keywords:** Recurrent venous thromboembolism, Biomarker, MicroRNA, Risk

## Abstract

**Background:**

Patients with unprovoked first venous thromboembolism (VTE) are at a high risk of recurrence. Although circulating microRNAs (miRNAs) have been found to be associated with VTE and are markers of hypercoagulability, this study is the first to examine whether circulating miRNAs are associated with the risk of VTE recurrence.

**Results:**

A nested case-control study design was used where plasma samples were obtained from 78 patients with unprovoked VTE from the Malmö Thrombophilia Study (MATS). A total of 39 VTE patients with recurrent VTE (cases) were matched with 39 VTE patients without recurrent VTE (controls) defined by age and sex (MATS population). Plasma levels of 179 different miRNAs were evaluated in the 78 samples (after anticoagulant treatment was stopped) using qPCR. A total of 110 miRNAs were detected in all samples. Among those, 12 miRNAs (miR-15b-5p, miR-106a-5p, miR-197-3p, miR-652-3p, miR-361-5p, miR-222-3p, miR-26b-5p, miR-532-5p, miR-27b-3p, miR-21-5p, miR-103a-3p, and miR-30c-5p) were found to be associated with recurrent VTE after multiple correction test and conditional logistic regression analysis. A further analysis showed that miR-15b-5p, miR-197-3p, miR-27b-3p, and miR-30c-5p exhibited a trend over time, with a larger difference in miRNA levels between cases and controls for earlier recurrence. Of these 12 miRNAs, 8 miRNAs significantly correlated with circulating transforming growth factor β1/2 (TGFβ1/2). Three of them correlated with platelet count.

**Conclusion:**

We have identified 12 plasma miRNAs that may have the potential to serve as novel, non-invasive predictive biomarkers for VTE recurrence.

**Electronic supplementary material:**

The online version of this article (10.1186/s13148-019-0627-z) contains supplementary material, which is available to authorized users.

## Background

Venous thromboembolism (VTE) is the third most common cardiovascular disease with an estimated annual incidence rate of 100–200 events per 100,000 individuals [1–3]. The most severe manifestation of VTE is fatal pulmonary embolism (PE) with an annual incidence rate of 2–4 events per 100,000 individuals [4]. VTE is a chronic disease with a recurrence risk of up to 20% within 3 years [5]. Recurrent VTE can be fatal in 10–20% of cases [6]. Patients with an unprovoked VTE (without an identified major clinical risk factor such as recent surgery or trauma, female hormone therapy or pregnancy, or malignancies) have a higher risk of recurrence after discontinuation of anticoagulation treatment compared with those patients with a provoked VTE [5, 7]. Long-term anticoagulation treatment, after the initial VTE, may prevent the recurrence but extended anticoagulation treatment may increase the risk of bleeding complications including fatal intracranial hemorrhage [6, 8]. Therefore, it is important to identify the patients, with a high risk of recurrence, who would benefit most from continuous anticoagulant treatment. There are a number of identified risk factors related to recurrent VTE, such as unprovoked VTE, elevated D-dimer levels, male gender, obesity, thrombophilia, and family history of VTE [9–13]. There is now a consensus regarding the optimal duration of anticoagulation treatment after an unprovoked VTE event. The treatment choice is between a short duration of 3–6 months and an extended duration. Clinical prediction rules have been developed to aid in this choice but the ability of such rules to predict bleeding appears to be low [14, 15]. Thus, better novel biomarkers for prediction of recurrent VTE risk could be of value.

MicroRNAs (miRNAs) are short, endogenous, and non-coding single-stranded RNAs that inhibit gene expression by promoting messenger-RNA (mRNA) degradation or inhibiting translation [16]. miRNAs have been shown to play essential roles in various biological processes during development and tissue homeostasis by regulating the expression of approximately 90% of all human genes [17]. The majority of miRNAs are expressed intracellularly. However, numerous miRNAs have been detected in the extracellular space, including blood and other body fluids [18]. Circulating miRNAs can be secreted from cells into the blood in different ways, e.g., enclosed in exosomes or associated with proteins [19, 20]. They are resistant to nuclease digestion and can be measured reproducibly, which makes circulating miRNAs attractive as potential biomarkers for diseases. Over the past decade, circulating miRNAs, as potential biomarkers, have been documented in many diseases such as cancer, psychiatric diseases, diabetes mellitus, and heart failure [21–25]. However, there is only limited evidence on potentially altered circulating miRNA levels in VTE. To our knowledge, only a few studies have investigated the association between circulating miRNAs and unprovoked VTE [25–29]. Wang et al. found that miR-424-5p is increased in patients with acute venous thrombosis and that it is correlated with a marker of hypercoagulability (D-dimer and APC-PCI complex) [25]. Thus far, however, no results regarding miRNA expression in recurrent VTE patients have been reported.

The present study used data from a prospective population-based study conducted in the south of Sweden; Malmö Thrombophilia Study (MATS) [30, 31]. In this study, the expression of plasma miRNAs was measured in 39 patients with recurrent VTE (cases) and 39 with non-recurrent VTE (controls) 2 weeks after discontinuation of anticoagulation. We hypothesized that specific miRNAs expression profile could be used to distinguish patients at high and low risk of recurrence. Therefore, our aim was to investigate the association between circulating miRNAs and the risk of VTE recurrence.

## Results

### Patients’ characteristics

Baseline characteristics of the 78 participants are shown in Table 1. Cases were those who were diagnosed with recurrent VTE during the follow-up period, whereas controls were those without recurrent VTE. The mean age was 65.3 years for cases and 65.1 years for controls. A total of 67% of the participants with recurrent VTE had thrombophilia compared with 41% of the participants without recurrent VTE (*p* = 0.09). There was no significant difference between the two groups in body mass index (BMI), duration of anticoagulant treatment, smoking status, and family history, which was defined as a history of VTE in first-degree relatives (sibling, son/daughter, or parent).

**Table 1:** Baseline characteristics of the study population

	Cases (recurrent VTE) (*n* = 39)	Controls (non-recurrent VTE) (*n* = 39)	*p* value
Age at first VTE, years			
Median (IQR)	65.3 (11.7)	65.1 (11.9)	–
Sex, *n* (%)			
Male	23 (59)	23 (59)	–
Female	16 (41)	16 (41)	
BMI			
Median (IQR)	28.5 (7.6)	25.8 (7.4)	0.34^b^
Thrombophilia^a^, *n* (%)			
Yes	26 (67)	16 (41)	
No	13 (33)	21 (54)	0.09^c^
Duration of anticoagulation, days			
Median (IQR)	182 (97)	182 (13)	0.69^b^
Smoking, *n* (%)			
Yes	8 (21)	3 (8)	
Earlier	14 (36)	18 (46)	
Never	12 (31)	17 (44)	0.12^c^
Family history of VTE, *n* (%)			
Yes	11 (28)	8 (21)	0.25^c^
No	27 (69)	30 (77)	

*Abbreviations*: *BMI* = body mass index; *IQR* = interquartile range; *VTE* = venous thromboembolism

^a^Thrombophilia: factor V Leiden, factor II mutations, protein S, protein C, and antithrombin deficiency

^b^Tested by Wilcoxon signed-rank test

^c^Tested by conditional logistic regression

### miRNAs screening data

A total of 179 miRNAs were analyzed in all 78 samples. Additional file 1: Figure S1 shows the sample quality control using spike-ins, UniSp2/UniSp4/UniSp5/ UniSp6, and UniSp3 (technical controls). The steady level of these assays shown in the graphs indicates that extraction, reverse transcription, and qPCR were successful. In addition, no hemolysis samples were present in the study population as measured by the ratio of miR-451a to miR-23a-3p. Of the 179 miRNAs analyzed in all 78 samples, an average of 150 miRNAs were detectable in more than 90% of the samples, and 110 miRNAs were detected in all samples.

### miRNAs profiling association with VTE recurrence

After further analysis of these 110 miRNAs (as shown in Additional file 2: Table S1), we found that 14 miRNAs were expressed in all samples and were significantly different (after adjusting for multiple testing using Benjamini-Hochberg correction with a false discovery rate (FDR) of 25%) [32] between cases and controls (presented in Table 2): miR-15b-5p, miR-106a-5p, miR-197-3p, miR-652-3p, miR-361-5p, miR-222-3p, miR-26b-5p, miR-532-5p, miR-27b-3p, miR-21-5p, miR-103a-3p, miR-30c-5p, miR-146b-5p, and miR-22-3p. Of these 14 differentially expressed miRNAs, miR-15b-5p, miR-222-3p, miR-26b-5p, miR-532-5p, miR-21-5p, miR-30c-5p, miR-146b-5p, and miR-22-3p were increased in cases compared to controls, whereas miR-106a-5p, miR-197-3p, miR-652-3p, miR-361-5p, miR-27b-3p, and miR-103a-3p were decreased (Additional file 1: Figure S2).

**Table 2:** Differentially expressed miRNAs in cases and controls in recurrent VTE

miRNAs^a^, ΔCt^b^ Median (IQR)	Cases (recurrent VTE) (*n* = 39)	Controls (non-recurrent VTE) (*n* = 39)	*p* value
miR-15b-5p	− 1.13 (0.49)	− 1.42 (0.53)	0.00005
miR-106a-5p	2.93 (0.30)	3.2 (0.37)	0.0006
miR-197-3p	− 1.62 (0.51)	− 1.30 (0.85)	0.0009
miR-652-3p	0.08 (0.62)	0.26 (0.63)	0.003
miR-361-5p	− 0.32 (0.56)	− 0.25 (0.37)	0.004
miR-222-3p	0.78 (0.45)	0.72 (0.50)	0.009
miR-26b-5p*	− 1.07 (0.44)	− 1.19 (0.47)	0.016
miR-532-5p	− 2.50 (0.66)	− 2.95 (1.04)	0.018
miR-27b-3p	1.49 (0.43)	1.72 (0.59)	0.019
miR-21-5p*	3.77 (0.34)	3.64 (0.29)	0.021
miR-103a-3p	2.72 (0.53)	2.85 (0.56)	0.022
miR-30c-5p*	− 0.43 (0.62)	− 0.47 (0.58)	0.023
miR-146b-5p	− 3.69 (1.02)	− 4.19 (0.90)	0.024
miR-22-3p	1.11 (0.40)	0.98 (0.42)	0.024

*Abbreviations*: *IQR* = interquartile range

^a^All miRNAs shown were significant after adjusting for the false discovery rate using the Benjamini-Hochberg correction. False discovery rate was chosen to 0.25 (25% false positives are allowed)

^b^*ΔCt* = Ct_global mean_ − Ct_miR of interest_

*Paired *t* tests were used instead of Wilcoxon signed rank test

To investigate the association between circulating miRNAs and the risk of VTE recurrence, we performed conditional logistic regression analysis on the 14 miRNAs displayed in Table 2. Twelve of the 14 miRNAs were significantly associated with the risk of VTE recurrence (Table 3). Two miRNAs, miR-146b-5p and miR-22-3p, were not significantly associated with recurrent VTE. The odds of having recurrent VTE were increased (OR > 1) for a one standard deviation (SD) increment in miRNA levels for miR-15b-5p, miR-222-3p, miR-26b-5p, miR-532-5p, miR-21-5p, and miR-30c-5p. By contrast, the odds decreased (OR < 1) for miR-106a-5p, miR-197-3p, miR-652-3p, miR-361-5p, miR-27b-3p, and miR-103a-3p (Table 3). The highest OR was for miR-15b-5p (OR = 7.8).

**Table 3:** Risk estimates for recurrent VTE based on single miRNA levels^a^

miRNAs^b^	OR	*p* value^c^	95% CI
miR-15b-5p	7.8	0.003	2.1; 29.3
miR-106a-5p	0.37	0.004	0.19; 0.72
miR-197-3p	0.40	0.006	0.21; 0.77
miR-652-3p	0.40	0.009	0.20; 0.79
miR-361-5p	0.41	0.01	0.21; 0.83
miR-222-3p	2.4	0.02	1.18; 4.93
miR-26b-5p	1.96	0.03	1.08; 3.55
miR-532-5p	2.7	0.04	1.04; 7.05
miR-27b-3p	0.57	0.03	0.34; 0.96
miR-21-5p	1.91	0.04	1.05; 3.48
miR-103a-3p	0.45	0.02	0.22; 0.90
miR-30c-5p	1.75	0.04	1.03; 2.97
miR-146b-5p	1.56	0.053	0.99;2.44
miR-22-3p	0.58	0.07	0.32;1.05

^a^*ΔCt* = Ct_global mean_ − Ct_miR of interest_

^b^All miRNAs were standardized (by standard deviation = SD). Odds ratios (ORs) are expressed per one SD increment. Only significant miRNAs (*p* value < 0.05) are shown

^c^Conditional logistic regression

**Fig. 1:**
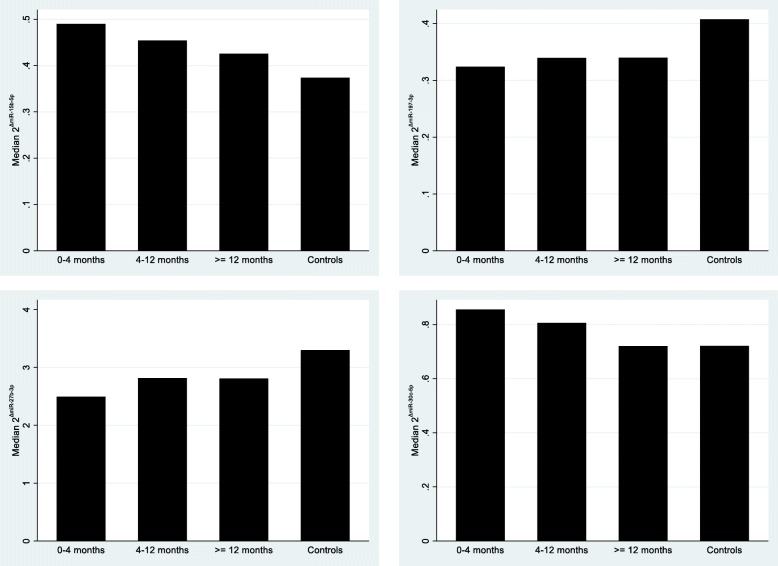
The expression levels of the 12 identified most significant miRNAs regarding time to recurrent VTE during follow-up

We further analyzed the expression levels of the 12 most significant miRNAs regarding time to recurrent VTE during follow-up. As shown in Fig. 1, the following miRNAs, miR-15b-5p, miR-197-3p, miR-27b-3p, and miR-30c-5p exhibited a trend over time, with larger difference in miRNA levels between cases and controls for earlier recurrence (*P*_trend_ < 0.05). The other miRNAs showed no significant pattern of this time-dependency (data not shown).

### In silico search for miRNA target genes and biological pathways

In order to further investigate the pathophysiological roles of miRNAs in VTE recurrence, we performed an in silico search to identify putative targets and pathways. We found that these miRNAs potentially regulated important genes, such as the human genes encoding for vascular endothelial growth factor (VEGF), metalloproteases, cytokines, and protein kinases (MAPK) that participate in the regulation of activation of monocytes, platelets, and endothelial cells (Table 4).

**Table 4:** Global miRNA target analysis for the 12 identified most significant miRNAs

miRNA name	Number of predicted target genes^a^	Validated target genes^b^
hsa-miR-15b-5p	1178	CCNE1, RECK, BCL2, CCND1, IFNG, CHEK1, SMAD7, FOXO1, SMURF1, CRIM1, FGF2, WEE1, FUT2, KDR, HNF1A, AKT3, AGO2, INSR, CCND3, SDCS3, RAB1A, MMP9, RIM14, MTSS1, PEBP4, OIP5, VEGFA, EIF4A1, AXIN2, PURA
hsa-miR-106a-5p	1072	E2F1, VEGFA, TGFBR2, CDKN1A, HIPK3, MYLIP, RB1, APP, RUNX1, ARID4B, VEGFA, IL10, FAS, CYP19A1, PAK5, PTEN, SIRPA, SLC2A3, BMP2, STAT3, CCND1, ATM, RUNX3, TIMP2, MAPK9, LIMK1, FASTK, MAPK14, ULK1, HIF1A, TP53, MSH3, APC, CXCL8, ATG7, IL1B, IL6, TGFB1, TBC1D9, CDX2, MGST2, ERCC1, RND3, RARB, HMGA2, HIPK3, MYLIP, ARID4B, VEGFA, CYP19A1, SIRPA, CASP7, MYB, MFN2
hsa-miR-197-3p	410	TUSC2, NSUN5, CD82, BMF, PMAIP1, MTHFD1, FOXJ2, MAPK1, RAN, TSPAN3, ACVR1
hsa-miR-652-3p	170	LLGL1, ZEB1
hsa-miR-361-5p	298	VEGFA, STAT6, SND1, TWIST1
hsa-miR-222-3p	433	STAT5A, CDKN1B, SOD2, MMP1, FOXO3, CDKN1C, KIT, TMED7, ETS1, PPP2R2A, TIMP3, DIRAS3, FOS, ESR1, BBC3, PTEN, SSSCA1, RECK, TRPS1, CERS2, SSX2IP, DKK2, PHACTR4, rf25, INPP4B, ZFAND5, FAM214A, LYPLA1, TIPARP, TP53BP2, MEGF9, VGLL4, GNAI3, GAS5, PRDM1, GNAI2, SMAD5, RUNX2, FOXO1, BMF, PLXNC1, BCL2L11, DICER1, TNFSF10, ICAM1, SELE, TP53, CORO1A, TCEAL1, DICER1, GRB10, ARID1A, ADAM1A
hsa-miR-26b-5p	1879	SERBP1, PTGS2, EPHA2, ABCA1, ARL4C, GATA4, CHORDC1, NR2C2, TAB1, EZH2, USP9X, KPNA2, RB1, NAMPT, PTEN, COX2, COL1A2, CTGF, TLR4, ST8SIA4, PDE4A, SOCS6, FH, HGF, LARP1, SERBP1, CDK6, CCNE1, PLOD2, IGFR1, MIEN1, ULK2, SMAD1, HAS2, IGF1, JAG1
hsa-miR-532-5p	94	RUNX3, TERT, NKD1, FASN, SYK, TRAPPC2B
hsa-miR-27b-3p	1043	CCNT1, WEE1, ST14, MMP13, ADORA2B, CYP1B1, PPARG, EDNRA, EYA4, VDR, SEMA6A, VEGFC, CREB1, ABCA1, PSAP, LDLR, WNK1, ENDOU, BNIP3, RMND5A, CRISP2, LPIN1, CCNYL1, CAB39L, CPPED1, CNN3, FOXO1, NR2F2, NR5A2, ROR1, CCNG1, FZD7, OSBPL6, CDH11, EGFR, MET, CX3CL1, SOCS6, UCA1, PINK1, NOTCH1, PAX3, CYP3A4, TRAPPC2B, KHSRP, PAX7
hsa-miR-21-5p	644	TGFBR2, TGBR3, TGFB1, RASGRP1, CDC25A, BCK2, RPS7, JAG1, SMRCA4, SPRY2, DUSP10, TIMP3, SOX5, MTAP, DOCK7, DOCK5, RECK, PIAS3, E2F2, PTEN, E2F1, LRRFIP1, CCL20, TPM1, NFIB, APAF1, BTG2, HIPK3, PDCD4, RHOB, ANP32A, SERPINB5, BMPR2, RASA1, MYC, ERBB2, JMY, TOPORS, HNRNPK, DAXX, TP53BP2, TP63, PPIF, MSH2, MSH6, TIAM1, ISCU, EIF4A2, ANKRD46, IL1B, ICAM1, PLAT, CDK2AP1, DOCK4, PPARA, NTF3, COL4A1, FASLG, SMAD7, SOX2, RMND5A, MMP2, VEGFA, SASH1, SERPINI1, DDAH1, PIK3R1, MMP9, ELAVL4, PTPN14, TOR1AIP2, PELI1, YOD1, STAT3, SATB1, WWP1, HPGD, MYD88, IRAK1, VHL, GDF5, IL12A, SECISBP2L, REFL, CXCL10, GAS5, RHO, CASC2, DNM1L, STUB1, LRP6, PSMD9
hsa-miR-103a-3p	780	CAV1, CCNE1, CDK2, CREB1, DICER1, KLF4, CYPC8, ID2, CDK6, MYB, SNCG, OPRM1, AGO1, GPRC5A, SERPINB5, MEF2D, SFRP4, OLFM4, PIEZO1, ADAM10, RUNX2, BNIP3, CACNA1C, GPD1, DAPK1, PTEN, TIMP3, MYCN
hsa-miR-30c-5p	1149	MUC17, UBE2I, SERPINE1, SNAI1, HSPA4, TGIF2, HDAC4, SOCS3, CUL2, NEDD4, SOCS1, ITGB3, ARHGEF6, ITGA4, PIK3R2, MATA1, IL11, DDIT4, DLL4, BCL9, IDH1, RARB, NCOR2, RFX6, RUNX2, CASP3, NOTCH1, TP53, BECN1, MED23, CAMK2D, IER2, CDC42, PAK1, FASN, FOXO3, CTGF, SMAD1, VIM, TWF1, MTTP, SNAI2, EIF2S1, RASAL2

^a^MiRSystem (http://mirsystem.cgm.ntu.edu.tw/), MiRTarBase (http://mirtarbase.mbc.nctu.edu.tw), and miRDB, version 4.0 (http://mirdb.org)

^b^Validated with western blot, qPCR, and/or reporter assay

We then used the DAVID annotation tool for the pathway analysis (DAVID 6.8, https://david.ncifcrf.gov/), and the most important pathways potentially involved in VTE pathophysiology are listed in Table 5, such as phosphatidylinositol 3-kinase-protein kinase B (PI3Ks-Akt), mitogen-activated protein kinase (MAPK), TGF-beta, Hippo signaling pathway, and hypoxia-inducible factor (HIF). The full list of biological pathways potentially regulated by these miRNAs is shown in Additional file 3: Table S2.

**Table 5:** Summary of KEGG pathway annotation of the 12 identified most significant miRNA targets (DAVID 6.8)^a^

Pathways	Genes involved in the pathway	*p* value^b^
Pathways in cancer (JAK-STAT pathway and ERK signaling pathway) [38]	69	1.4E-24
TNF signaling pathway [70]	26	5.4E-12
PI3K-Akt signaling pathway [40]	41	8.5E-9
Hippo signaling pathway [43]	26	1.3E-8
TGF-beta signaling pathway [31]	19	3.2E-8
HIF-1 signaling pathway [42]	21	1.3E-8
Focal adhesion [71]	27	9.0E-7
MAPK signaling pathway [41]	26	1.1E-4
Rap1 signaling pathway [39]	23	1.2E-4

^a^All the validated predicted miRNA targets (genes listed on Table 4) were run KEGG pathway annotation using the DAVID gene annotation tool

^b^Fisher’s exact *p* value after Benjamini-Hochberg correction (https://david.ncifcrf.gov/)

### Correlation of miRNAs associated with recurrent VTE and TGFβ1/2/3 and plasma clot parameters

We previously reported that there was an association between higher risks of recurrent VTE and lower levels of plasma TGFβ1/2 [31]. In the present study, we found that there were correlations between TGFβ1 and most of the significant miRNAs (Table 6). The expression of miR-15b-5p, miR-106a-5p, miR-197-3p, miR-652-3p, miR-361-5p, miR-27b-3p, and miR-103a-3p were positively correlated with TGFβ1 expression, whereas miR-532-5p was negatively correlated. Similar results were also found for TGFβ2, but only miR-15b-5p, miR-197-3p, and miR-27b-3p correlated with TGFβ3 (data not shown). Thus, a total of 8 out of 12 miRNAs were correlated with TGFβ1/2 expression.

**Table 6:** Correlation of TGFβ1 and miRNAs associated with recurrent VTE

miRNA name	Correlation coefficient	*p* value	Correlation^a^
*miR-15b-5p*	0.26	*0.04*	Positive
*miR-106a-5p*	0.29	*0.01*	Positive
*miR-197-3p*	0.29	*0.02*	Positive
*miR-652-3p*	0.52	*< 0.001*	Positive
*miR-361-5p*	0.28	*0.02*	Positive
miR-222-3p	− 0.13	0.28	Negative
miR-26b-5p	− 0.09	0.46	Negative
*miR-532-5p*	− 0.3	*0.01*	Negative
*miR-27b-3p*	0.33	*0.006*	Positive
miR-21-5p	0.09	0.46	Positive
*miR-103a-3p*	0.26	*0.03*	Positive
miR-30c-5p	− 0.005	0.96	Negative

^a^Spearman’s rank correlation analysis

miRNAs in italics are significantly correlated with TGFβ1

Platelet count was positively correlated with the expression of plasma miR-652-3p (*r* = 0.31, *p* = 0.006). In addition, miR-197-3p (*r* = 0.23, *p* = 0.05) and miR-27b-3p (R = 0.21, *p* = 0.066) were also positively correlated with platelet count, but the correlations were not statistically significant. Some of these 12 identified miRNAs were also correlated with plasma clot parameters: miR-15b-5p (*r* = 0.34, *p* = 0.003), miR-652-3p (*r* = 0.26, *p* = 0.02), and miR-103a-3p (*r* = 0.32, *p* = 0.005) were positively correlated with the international normalized ratio (INR) and miR-26b-5p was negatively correlated with both protein C and protein S (Additional file 4: Table S3).

### The potential role of these miRNAs in primary VTE

We also checked whether the 12 miRNAs profile associated with recurrent VTE identified in the present study was also associated with primary VTE in the SCORE cohort. None of these miRNAs were significantly associated with primary VTE (data not shown).

## Discussion

As far as we know, this is the first study to explore the association of circulating miRNAs and the risk of VTE recurrence. We found that expression of miR-15b-5p, miR-106a-5p, miR-197-3p, miR-652-3p, miR-361-5p, miR-222-3p, miR-26b-5p, miR-532-5p, miR-27b-3p, miR-21-5p, miR-103a-3p, and miR-30c-5p were significantly different between cases (recurrent VTE) and controls (non-recurrent). High plasma levels of miR-15b-5p, miR-222-3p, miR-26b-5p, miR-532-5p, miR-21-5p, and miR-30c-5p and lower levels of miR-106a-5p, miR-197-3p, miR-652-3p, miR-361-5p, miR-27b-3p, and miR-103a-3p were significantly associated with risk of VTE recurrence. Moreover, miR-15b-5p, miR-197-3p, miR-27b-3p, and miR-30c-5p exhibited a trend over time, with a larger difference in miRNA levels between cases and controls for earlier recurrence. In addition, we found that some of the miRNAs were correlated with plasma TGFβ levels, platelet count, and plasma clot properties.

In particular, among patients with unprovoked VTE, identifying patients who are at highest risk for recurrent VTE is a clinically important issue. At present, a few biomarkers such as D-dimer and endogenous thrombin potential (ETP), circulating P-selectin, plasma TGFβ levels, and plasma clot properties have been suggested to identify VTE patients that are at risk of VTE recurrence [31, 33–36]. However, it still remains a challenge to predict individuals at risk of VTE recurrence [37]. The present study shows that miRNAs are a novel group of biomarkers that are potentially useful for predicting the risk of VTE recurrence.

By performing an in silico search for miRNA target genes and pathway enrichment analysis, we found, as expected, that miRNAs could regulate different genes and are involved in different pathways. However, the most important pathways, which could have a role in the pathophysiology of VTE, are discussed here; MAPK signaling pathways have been previously shown to be involved in the pathophysiology of thrombosis or platelet activity in a direct or indirect manner [31, 38–42]. Similarly, the Hippo signaling pathway has been shown to play an important role in megakaryocyte differentiation and platelet production [41, 43]. HIF-1 pathway is known to promote endothelial permeability and activation, coagulopathy, and recruitment of inflammatory cells, and contribute to the initiation or development of venous thrombosis through its target genes [42]. In each pathway, the function of miRNAs overlaps with each other. For example, all the 12 identified miRNAs involved in the PI3K-Akt signaling pathway, which could regulate platelet activation in thrombus formation and stabilization [40]. These analyses further illustrated the possible roles and mechanisms of these differentially expressed miRNAs in VTE.

Our previous study concerning VTE recurrence showed that lower plasma levels of TGFβ1/2 were significantly associated with higher risks of recurrent VTE [31]. In the present study, we found that plasma levels of miR-15b-5p, miR-106a-5p, miR-197-3p, miR-652-3p, miR-361-5p, miR-27b-3p, and miR-103a-3p were positively correlated with TGFβ1/2 expression, whereas miR-532-5p were negatively correlated. The miR-15 family was identified as a novel regulator of cardiac hypertrophy and fibrosis acting by inhibiting the TGFβ-pathway [44]. MiR-27b and miR-532 were also demonstrated to be involved directly or indirectly in the TGFβ pathway in animal experiments [45, 46]. Based on these observations, it is possible that the role of these miRNAs in VTE recurrence may also be TGFβ-pathway-dependent. However, this needs to be investigated in future studies.

Another important finding of this study is that miRNAs were correlated with plasma clot properties. For example, miR-15b-5p, miR-652-3p, and miR-103a-3p were positively correlated with the INR while plasma miR-197-3p, miR-27b-3p, and miR-652-3p were correlated with platelet count. Nordstrom et al. found that subtherapeutic INR levels were associated with a more than threefold increased risk of VTE recurrence and low platelet counts also predicted a greater risk of VTE recurrence [47].

There are important differences in risk factor profiles between primary and recurrent VTE [48]. Therefore, we tested these 12 miRNAs in a primary VTE population—the SCORE study [25]. However, none of these miRNAs were significantly associated with primary VTE [28]. As VTE encompasses both DVT and PE, the different miRNA expression profiles between these studies could be related to the differences in the subsets of selected patient cohorts or duration after initial thrombotic event. Therefore, the results from the present study do not allow us to conclude that miRNAs identified in recurrent VTE were not related to primary VTE. However, our finding of different miRNA profile for primary and recurrent VTE is in line with a large number of studies that have shown that the risk factor profile is different between primary and recurrent VTE, as reviewed by Cannegieter and Van Hylckama Vlieg [48]. Thus, different miRNAs may play a different role in primary and recurrent VTE. A validation study therefore is warranted due to the high variation between studies.

Recent studies have suggested that arterial and venous thrombosis share common risk factors [49–51]. In the present study, we found that high levels of plasma miR-15b-5p, miR-222-3p, miR-26b-5p, miR-532-5p, miR-21-5p, and miR-30c-5p were significantly associated with a higher risk of VTE recurrence. Most of these miRNAs have been involved in other cardiovascular diseases. Tijsen et al. found that the miR-15 family (miR-15a/b, miR-16, miR-195, miR-497, and miR-322) is upregulated in cardiac hypertrophy and heart failure. Higher plasma levels of miR-21-5p were significantly associated with acute myocardial infarction (AMI) patients [52]. miR-222 has been reported to play an important role in physiological and pathological processes in the heart and may be a potential cardiovascular biomarker and a new therapeutic target in cardiovascular diseases [53]. Circulating miR-26b-5p and 30c-5p were also related to heart failure [54, 55]. In our analysis, low levels of miR-106a-5p, miR-197-3p and miR-652-3p, miR-361-5p, miR-27b-3p, and miR-103a-3p were associated with higher risk of VTE recurrence. Previous studies reported that low levels of miR-106a-5p and miR-197-3p were associated with a high risk of AMI [56, 57] and low levels of miR-106a-5p, miR-103, miR-361-5p, miR-27b-3p, and miR-652-3p were related to acute heart failure [54, 58–61]. Put together, there may be some specific miRNAs generally associated with both arterial and venous thrombosis.

This is a hypothesis-generating study in which, for the first time, the role of circulating miRNAs in VTE recurrence has been studied. Our findings require an independent validation study. There are several general limitations in our experimental design that should be considered when interpreting our results. The main limitation of the study is the relatively small number of patients. Although we adjusted the results for multiple comparisons, we found several microRNAs related to recurrent VTE. A potential mechanistic limitation is that the cellular origin of these miRNAs could not be determined. Therefore, we are unable to detect the mechanism behind the changes of these miRNAs.

## Conclusion

Our results suggest that platelets and the TGFβ pathways regulating miRNAs may serve as novel biomarkers for VTE recurrence. The present study warrants further investigation on the role of miRNAs in recurrent VTE.

## Material and methods

### Study design and subjects

The present study used a nested case-control design, including selected subjects from a pre-existed cohort that has been followed over time. The subjects were selected from a prospective population-based cohort study at Skåne University Hospital in Sweden; Malmö Thrombophilia Study (MATS). MATS included a total of 1465 consecutive patients diagnosed with VTE between March 1998 and December 2008 [30]. The inclusion criteria in MATS were the following: age > 18, ability to speak and read Swedish, and an objective diagnosis of DVT or PE made with phlebography, duplex ultrasound, computed tomography (CT), lung scintigraphy, or magnetic resonance imaging (MRI) [30]. The following information was recorded: location of DVT, immobilization and cast therapy, hospitalization, surgical intervention, malignancies that were diagnosed previously or at diagnosis of VTE, use of contraceptive pills, hormonal therapy, pregnancy and postpartum period (first 6 weeks after delivery), family history of VTE (history of VTE in first-degree relatives), VTE recurrence during the follow-up period (until the end of the study in December 2008), and other risk factors, such as tobacco use or traveling information. A research nurse was responsible for screening the hospital records of patients. All VTE cases and recurrent events were objectively confirmed in this well-characterized population-based study. The participation rate was high (70%). The remaining 30% of patients did not participate in blood sampling, questionnaire, and complete risk factor analysis because of language problems, dementia, or other severe diseases [30].

According to the standard treatment protocol of Skåne University Hospital, all VTE patients were treated with low-molecular-weight heparin or unfractionated heparin during the initiation of oral anticoagulants (OAC, Warfarin). The treatment protocol recommended that 3–6 months OAC therapy for first-time VTE and consideration of longer-term therapy for recurrent VTE.

Thrombophilia was defined as presence of the factor V Leiden (FVL) mutation (rs6025) or factor II G20210A mutation (rs1799963), or a level below the laboratory reference range of protein C [< 0.7 k international unit (kIU/L)], free protein S (women < 0.5 kIU/L, men < 0.65 kIU/L) or antithrombin (< 0.82 kIU/L) in patients without warfarin treatment.

A total of 78 patients (26–85 years) with unprovoked VTE were recruited from MATS. Unprovoked VTE was defined as complete absence of provoking factors at the time of diagnosis. The following were regarded as provoking factors: malignancies, surgical intervention, use of contraceptive pills or hormonal therapy, pregnancy or puerperium, acute medical conditions (AMI, acute ischemic stroke, major infectious disease), and marked immobilization (bed rest > 3 days, patients in a wheelchair, long-distance travel ≥ 4 h within the last 14 days). Patients who had any of these provoking factors were excluded from the study (*n* = 283). Besides these exclusions, patients who had thrombotic events before inclusion (*n* = 25), or recurrence during anticoagulant therapy (*n* = 281), those who gave post-treatment samples during treatment (*n* = 382) and those who had inadequate or missing case report forms were also excluded (*n* = 51). Among the remaining 443 patients with unprovoked VTE, 41 suffered from recurrent VTE. Two plasma samples were excluded due to poor plasma quality (e.g., hemolysis). In total, 39 patients with recurrent VTE (cases) and 39 age-, sex-, and time of sampling matched patients from the MATS study population without recurrent VTE (controls) during the follow-up period were included in the present study.

The primary VTE cohort consisted of 238 (aged 16–95 years, 91 men and 147 women) plasma samples, 51 DVT patients and 183 patients without DVT (4 samples were excluded due to the low sample volume), selected from a prospective multicenter diagnostic management study (SCORE) [25].

### Sample preparation

The blood samples were taken 2 weeks after discontinuing oral anticoagulant therapy given to the patients with a first thrombotic event. Venous blood samples were collected into sodium citrate (3.8%)-treated tubes and were centrifuged at 2000×*g* for 15 min at 4 °C to obtain platelet-poor plasma. Samples were transferred to new RNase/DNase-free tubes and then were centrifuged again at 3000×*g*, 10 min at 4 °C to obtain platelet-poor plasma, the plasma was then aliquoted and stored at − 80 °C before further processing.

### Circulating miRNA exploration

Total RNA was isolated from 230 μl of plasma using the Qiagen miRNeasy Mini Kit (Qiagen GmbH, Hilden, Germany) according to the manufacturer’s protocol, with minor modifications. miRNAs were reverse transcribed using a Universal cDNA Synthesis kit (Exiqon, Vedbæk, Denmark). The resulting reverse transcription reaction product was stored at − 20 °C for further analysis. A detailed description of the methodology has been provided in previous articles [24, 62]. It is important for any types of qPCR experiments to ensure that the quality of the input RNA is sufficiently high for effective amplification. We used spike-in RNAs in all steps of our experiments, i.e., in the RNA extraction, the cDNA synthesis. RNA spike-in kit (Exiqon, Vedbæk, Denmark) for quality control of the RNA isolation (UniSp2/UniSp4/UniSp5) and cDNA synthesis (UniSp6) was applied.

For the initial screening, miRNA expression was screened using a Serum/Plasma Focus microRNA PCR Panel (Exiqon) comprising 179 LNA™ microRNA primer sets focusing on serum/plasma-relevant human miRNAs. A negative control without template from the reverse transcription reaction was included in the analyses. Quantitative real-time PCR (qPCR) was conducted using a CFX384 Real-Time PCR Detection System (Bio-Rad). The panels contain assays for synthetic RNA (UniSp2/UniSp4//UniSp5/ UniSp6) in the RNA spike-in kit for controlling the RNA isolation and cDNA synthesis. In addition, an inter-plate control (UniSp3) was present in all panels. Two hemolysis miRNAs (miR-23a-3p, miR-451a) were also included in the panels. A hemolysis index, the ratio of miR-451a to miR-23a (ΔCt = Ct _miR-23a-3p_ − Ct _miR-451a_) was used based on the qPCR detection according to a previous report [63]. Only the samples with ΔCT value < 7 were used in the following analysis. Undetectable data were assigned a default threshold cycle (Ct) value of 37. As there is no current consensus as to an appropriate reference miRNA for the normalization of plasma miRNAs in the qPCR analysis, we used the “global mean” as a reference in the analysis. The global mean is calculated as an average of all the detected assays in each sample. This means that the data set is not normalized to a “constant” but rather to the average expression for each sample. This normalization approach is used as a standard for big qPCR profiling projects [64]. We also normalized the 110 miRNAs with some endogenous miRNAs as normalizers. All the detected 110 miRNAs were compared and ranked using the currently available major computational programs (geNorm, Normfinder, BestKeeper, and the comparative ΔCt method) [65]. miR-423-5p, let-7i-5p, and miR-30e-5p were stable and abundant in the plasma, with a coefficient of variation of 1.8%, 2.0%, and 2.0% respectively. We therefore normalized 110 miRNAs with the geometric mean of miR-423-5p, let-7i-5p, and miR-30e-5p. The results were similar to that when using the global mean for normalization (data not shown). Ct values were then normalized to global mean using the following equation: ΔCt _miR of interest_ = Ct _global mean_ − Ct _miR of interest_. Relative expression of miRNAs was calculated with 2^ΔCt^. Fold changes were calculated as Fold change = 2^ΔCt cases –ΔCt controls^.

### miRNAs target analysis

The analysis of miRNA-predicted targets was performed using three different algorithms (miRSystem, version 20120229; miRTarBase 7.0, and miRDB, version 4.0) available for free academic use [66–68]. The targeted genes, which have been validated by qPCR, western blot, or reporter assay, were summarized in a table as gene symbols (Table 4). The gene ontology (GO) and Kyoto Encyclopedia of Genes and Genomes (KEGG) pathway analysis of these target genes were then performed by DAVID Bioinformatics Resources 6.8 (https://david.ncifcrf.gov/) [29, 69].

### Statistical analysis

Differences in sample characteristics between cases and controls were tested using Wilcoxon signed-rank test (for continuous variables) and conditional logistic regression (for the categorical variables).

To examine possible associations between miRNAs and recurrent VTE, we first tested the difference in ΔCt (Ct_global mean_ − Ct_miR of interest_) of all 110 miRNAs between cases and controls using Wilcoxon signed-rank test (for three miRNAs a paired *t* test could be used instead because it was normally distributed). We adjusted for the false discovery rate using the Benjamini-Hochberg correction. False discovery rate was set at 0.25 (25% false positives are allowed). The association was also examined by graphing mean and 95% CI of fold changes (2^ΔCt cases − ΔCt controls)^).

Thereafter, we used conditional logistic regression on the significant miRNAs from the analysis above (scaled to have the same standard deviation) to examine the effect of miRNAs on the risk for recurrent VTE, as estimated by odds ratios. In addition, we examined if time to recurrent VTE was associated with miRNAs. We divided the time to recurrent VTE into categories with a similar number of cases (0–4 months, 4–12 months, > = 12 months). For those miRNAs that showed a significant trend in a regression model, we presented the median values of relative expression of miRNAs (2^ΔCt^) in a bar plot per time interval and for controls.

Spearman’s correlation or Pearson’s correlation analysis was applied to the significant miRNAs and TGFβ, platelets count, and plasma clot parameters. The difference of miRNAs expression levels between primary VTE and non-VTE was tested by *t* test.

STATA version 14 (StataCorp LP) was used for all statistical analyses.

## Additional files

Additional file 1: **Figure S1.** The graph shows the raw Ct values for the control assays on all the samples (*n* = 78). Figure S2 Differential expression of 14 miRNAs in cases compared to controls significant after adjusting for the false discovery rate using the Benjamini-Hochberg correction. Fold changes (Fold change = 2^ΔCt cases − ΔCt controls^) with 95% CI. (PDF 282 kb)

Additional file 2: **Table S1.** Presentation of the 110 miRNAs (ΔCt = Ct_global mean_ − Ct_miR of interest_) in cases and controls. (XLSX 17 kb)

Additional file 3: **Table S2.** Eighty-two KEGG pathway annotation of the miRNA Targets (DAVID 6.8). (XLSX 15 kb)

Additional file 4: **Table S3.** Correlation between plasma clot parameters and miRNAs associated with recurrent VTE (*n* = 75). (DOC 45 kb)

## Abbreviations

BMI: Body mass index
CI: Confidence interval
DVT: Deep-vein thrombosis
IQR: Interquartile range
miRNAs: MicroRNAs
PE: Pulmonary embolism
SD: Standard deviation
TGFβ: Transforming growth factor
VTE: Venous thromboembolism
