# Supplementary material for: Association of recurrent venous thromboembolism and circulating microRNAs
Source: Clin Epigenetics. 2019 Feb 13;11:28. doi: 10.1186/s13148-019-0627-z (PMC6374897; doi:10.1186/s13148-019-0627-z)
Supplement: Supplementary file 1 — Figure S1. The graph shows the raw Ct values for the control assays on all the samples (n = 78). Figure S2 Differential expression of 14 miRNAs in cases compared to controls significant after adjusting for the false discovery rate using the Benjamini-Hochberg correction. Fold changes (Fold change = 2ΔCt cases − ΔCt controls) with 95% CI. (PDF 282 kb) [file 13148_2019_627_MOESM1_ESM.pdf]

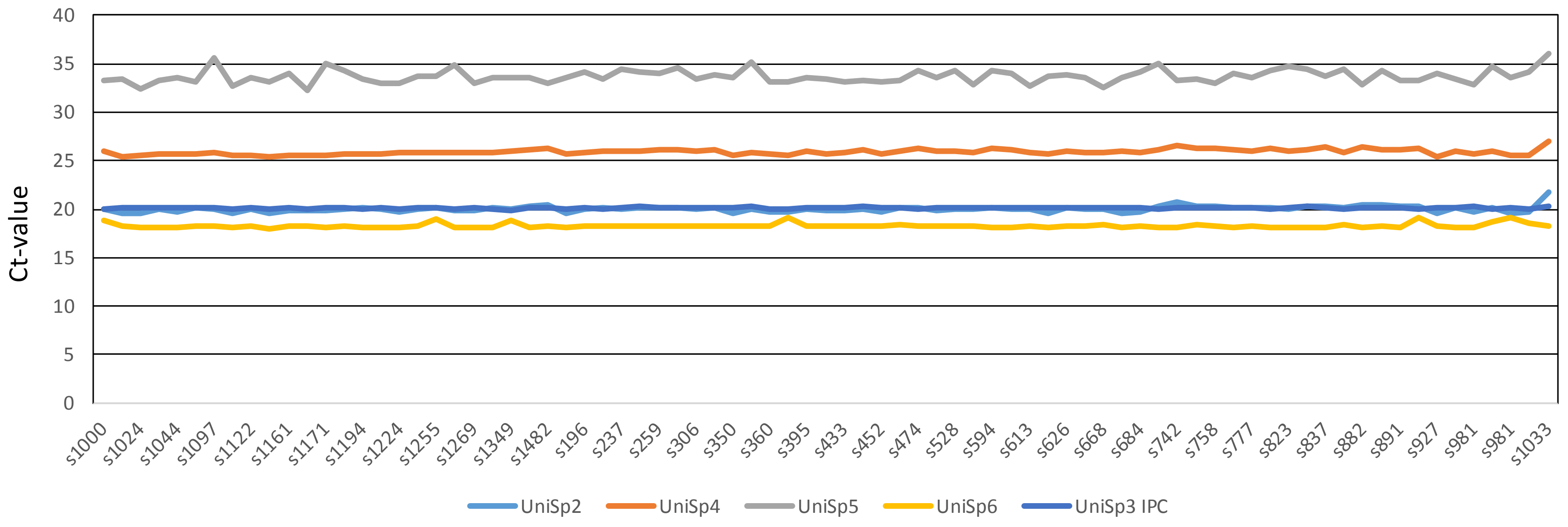

Figure S1. The graph show the raw Ct values for the control assays on all the samples (n=78)

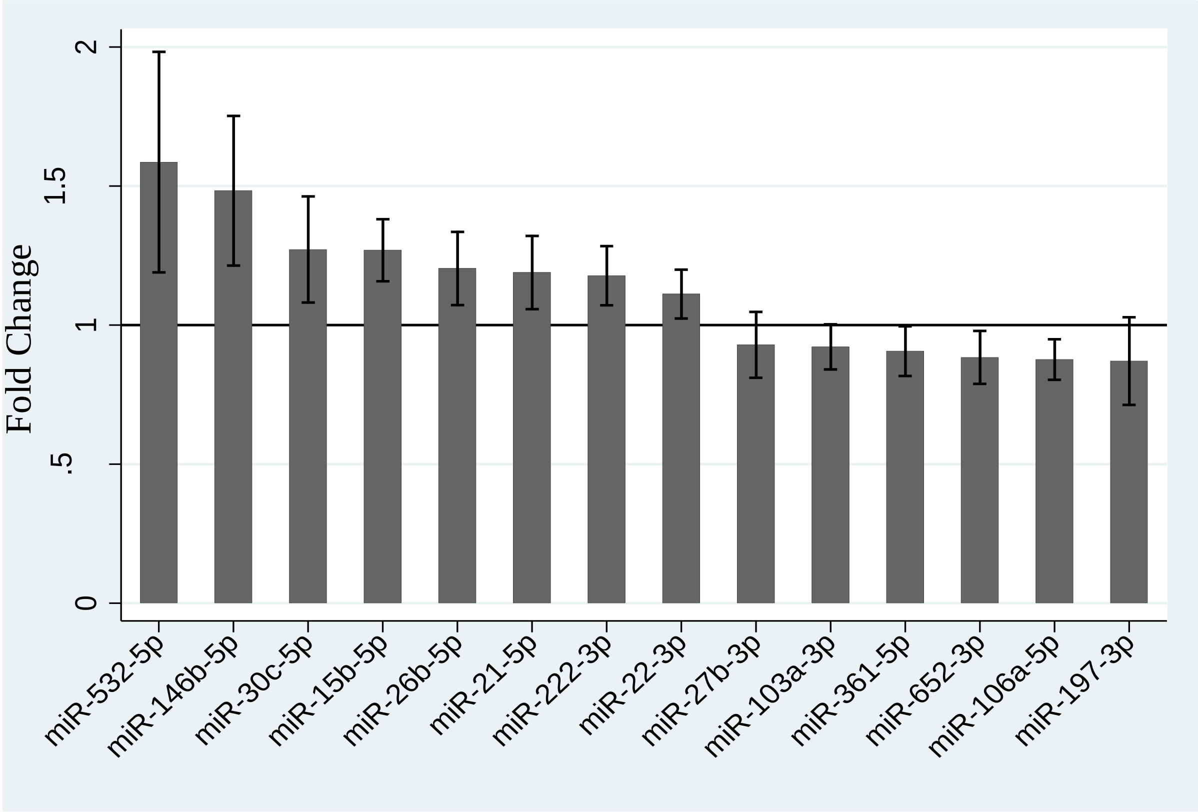

Figure S2. Differential expression of 14 miRNAs in cases compared to controls significant after adjusting for the false discovery rate using the Benjamini-Hochberg correction (**all p-values < 0.05 and showed in Table 2**). Fold Changes (Fold change=$2^{\Delta Ct\ cases - \Delta Ct\ control}$) with 95% CI.
