# Supplementary material for: Association of recurrent venous thromboembolism and circulating microRNAs
Source: Clin Epigenetics. 2019 Feb 13;11:28. doi: 10.1186/s13148-019-0627-z (PMC6374897; doi:10.1186/s13148-019-0627-z)
Supplement: Supplementary file 4 — Table S3. Correlation between plasma clot parameters and miRNAs associated with recurrent VTE (n = 75). (DOC 45 kb) [file 13148_2019_627_MOESM4_ESM.doc]

| **Table S3**. Correlation between plasma clot parameters and miRNAs associated with recurrent VTE (n=75) | | | | | | | | | | |
| --- | --- | --- | --- | --- | --- | --- | --- | --- | --- | --- |
|  | Platelet count^a^ | | Protein C^a^ | | Protein S^a^ | | INR^b^ | | Antithrombin^a^ | |
| miRNAs | r | P-value | r | P-value | r | P-value | r | P-value | r | P-value |
| miR-15b-5p | 0.14 | 0.24 | -0.18 | 0.13 | -0.04 | 0.73 | 0.34 | **0.003** | -0.10 | 0.34 |
| miR-106a-5p | 0.07 | 0.53 | 0.03 | 0.77 | 0.04 | 0.72 | 0.09 | 0.44 | 0.005 | 0.97 |
| miR-197-3p | **0.23** | **0.05** | -0.07 | 0.56 | 0.07 | 0.56 | -0.01 | 0.90 | 0.002 | 0.98 |
| miR-652-3p | **0.31** | **0.006** | -0.10 | 0.42 | -0.10 | 0.55 | 0.26 | **0.02** | 0.01 | 0.90 |
| miR-361-5p | 0.15 | 0.19 | -0.36 | 0.76 | 0.00 | 0.99 | 0.16 | 0.17 | 0.02 | 0.84 |
| miR-222-3p | 0.13 | 0.25 | 0.09 | 0.42 | 0.001 | 0.99 | -0.08 | 0.47 | 0.02 | 0.87 |
| miR-26b-5p | 0.08 | 0.51 | **-0.23** | **0.04** | **-0.23** | **0.04** | 0.05 | 0.64 | -0.09 | 0.44 |
| miR-532-5p | -0.13 | 0.28 | -0.03 | 0.79 | 0.19 | 0.10 | -0.04 | 0.72 | 0.02 | 0.86 |
| miR-27b-3p | **0.21** | **0.066** | **-0.26** | **0.02** | -0.12 | 0.29 | 0.17 | 0.15 | -0.04 | 0.71 |
| miR-21-5p | 0.01 | 0.90 | 0.01 | 0.95 | -0.21 | 0.07 | -0.07 | 0.55 | 0.04 | 0.75 |
| miR-103a-3p | 0.08 | 0.47 | -0.15 | 0.21 | 0.10 | 0.39 | 0.32 | **0.005** | -0.07 | 0.55 |
| miR-30c-5p | 0.05 | 0.64 | -0.06 | 0.59 | 0.03 | 0.78 | 0.08 | 0.48 | -0.04 | 0.74 |

r: correlation coefficient

^a^ Pearson Correlation; ^b^ Spearman's rank correlation Correlation coefficient

INR: International Normalized Ratio
